# Numerical simulation of unsteady aerodynamic interactions of contra-rotating axial fan

**DOI:** 10.1371/journal.pone.0200510

**Published:** 2018-07-19

**Authors:** Hengxuan Luan, Liyuan Weng, Yuanzhong Luan

**Affiliations:** 1 Department of Mechanical and Electronic Engineering, Shandong University of Science and Technology, Taian, Shandong, China; 2 College of Geomatics,Shandong University of Science and Technology, Qingdao, Shandong, China; Virginia Tech, UNITED STATES

## Abstract

This paper describes the investigations performed to better understand unsteady effect that develop in a contra-rotating axial fan. More specifically, this study focuses on rotor-rotor interactions effects on unsteady characteristic and blade aerodynamic force. The investigation method is based on three-dimensional URANS simulations, in conjunction with SST turbulence model. At first, the experimental measurements are compared to evaluate ability of the numerical method in estimation of unsteady flows. The results show that rotor-rotor interaction in the contra-rotating fan played an important role in aerodynamic efficiency. Unsteady effect increased flow losses of rotor 1, but effectively inhibited flow losses of rotor 2. The inhibition effect was mainly caused by wake recovery effect of upstream wakes in the flow passage of rotor 2. Meanwhile, negative jet flow enhanced boundary layer energy of the blade of rotor 2, so that flow separation was postponed. Different configurations consider five sets of axial spacing dimensions. Specific survey of flows under the same operation conditions indicates that axial spacing is responsible for the unsteady interaction effect. The blade aerodynamics analysis shows that the influence of the downstream potential flow disturbance on rotor 1 is greater than the effect of the upstream wake on rotor 2.

## Introduction

The industry of turbomachinery keeps on bringing up the efficiency and reducing the environmental influences of turbomachinery. In this way, the risks of oil shortage can be relieved, while the requirements for more stringent legislation with regard to emitted pollutants can be satisfied. These demands have been accomplished by the continuous improvement of turbomachinery technology. With the unique design, the contra-rotating technology is able to increase the pressure ratio and reduce the weight of machine of a turbomachinery [1]. However, the flow characteristics which are uncomfortable naturally have retarded engineers from applying the technology of contra-rotation in the conventional turbomachinery design. [2,3]. Therefore, it requires a better understanding of the unsteady flow effects in the turbomachinery adopts contra-rotation technology.

In recent years, the contra-rotating axial fan/compressor arouse a greater interest that contra-rotation enables to simplify machine structure and improve machine performance. Sharma [4] had thoroughly studied the effects of various parameters on the performance of a low-speed contra-rotating compressor, such as the speed ratio of the two rotors, rotor stagger, pitch-to-chord ratio and axial spacing between the rotors. The research showed that the above parameters all have certain influence on the performance of the contra-rotating compressor, but the axial spacing had a strong influence on the compressor performance. Mistry and Pradeep [5] reported the effect of variation in axial spacing and rotor speed combinations on the performance of a contra-rotating axial fan. They clarified the axial spacing and rotor speed plays an important role in the terms of overall performance. Nouri [6] carried out an experimental study on the design of the contra-rotating fan, revealed that speed ratio had an important impact on the overall performance. Chen [7] investigated the effects of speed ratio and rotation speed of rotor upstream over downstream on the performance of a contra-rotating compressor. The aerodynamic performance and noise of a contra-rotating fan under different rotation speed and axial spacing were numerically investigated by Luan [8,9]. Despite all that, it is still needed to carry out investigations to gain a further insight into the flow feature in contra-rotating fan. There is limited information in literatures on the unsteady effect of rotor-rotor interaction process.

There is an unsteady flow between adjacent blade rows or between the rotor stages. This unsteady phenomenon leads to nonlinear aerodynamic forces and strong coupling effects, which can lead to complex interference phenomena [10–12]. Comparing with the conventional fan, the flow field between the two rows of rotors are lack of carding of the stator in the contra-rotating fan, which makes the whole flow field highly unsteady. Thus the unsteady flow field in contra-rotating fan deserves further study. However, considering the cost and difficulty of the experiment, research on the unsteady flow of contra-rotating fan is relatively rare. Meyer [13] used a hot-wire anemometer to measure unsteady velocity fields in two rows of rotors of the high-speed contra-rotating fan under 54% of the designed rotation speed. Analysis results show that the wake effect of the rotor 1 would extend to downward areas of rotor 2. Meanwhile, the blade passing frequency of rotor 2 existed in the spectrogram of the downstream area of rotor 1. This result directly indicates interaction effect existing between two rows of rotors. Shigemitsu [14] conducted a numerical simulation of a contra-rotating axial fan, and pointed out the existence of interference at the contra-rotating rotor stage by analyzed the pressure distribution of the flow field. Mao [15] performed an unsteady numerical simulation of rotor-rotor interaction generated by a contra-rotating axial compressor, whose main points was potential effects from the rear rotor have a more pronounced influence on the front rotor. However, Due to complicity of inter-stage flow of the contra-rotating fan, unsteady effect caused by rotor-rotor interaction is still unclear [16]. There is rare systematic research on propagation of wakes in the contra-rotating fan.

In wake propagation, wakes will be cut by the downstream blade into segments and will be stretched and twisted under influences of the pressure field gradient. It is a type of highly unsteady flowing. Key[17] researched wake transport in the multi-stage axial flow compressor. Results show that strong interference phenomena existed between wakes and mainstreams and between wakes and the boundary layer. Through numerical simulation, Zhao[18] made detailed research of wake oscillation. The CFD method which analyzes wakes is conducted in ways that computation meshes are generated according to the real blade and a flow equation is then solved. In view of accuracy of the computation, the method generally requires a lot of computation meshes and a huge amount of computation resources. The actuator disk model is a computation method which has very wide engineering applications and is used to analyze wakes. It combines the blade element theory with the CFD method, neglects the real geometric appearance of the blade and embodies effects of the blade on surrounding airflows by force of the actuator disk. Based on the actuator disk theory, Crasto[19] predicted acting relations between wakes of multiple wind machines. Castellani[20] computed wake flow fields of the wind machines. Research results show that effective information of wake effects can be given by the actuator disk theory. The method of actuator disk is advantageous in that the balance problem between computation resources and computation accuracy can be solved, while computation results can satisfy engineering application. However, the actuator disc method neglects the wall effect and simplifies the impeller into a disk. Hence, turbulence intensity generated from impeller rotation will be underestimated.

The present work aims to study the development and interaction process of the rotor-rotor interaction in a contra-rotating fan. To make up for the experimental or numerical investigation, for a contra-rotating axial fan, the unsteady characteristics of two-stage rotors were investigated in the present study. Rules of downward propagation of wakes were researched. Effects of rotor-rotor interaction on unsteady aerodynamic force were analyzed. Hence, the unsteady flow mechanism in the contra-rotating fan could be known profoundly. The effect of axial spacing is also investigated for the unsteady aerodynamic characteristics. This paper is organized as follows. The numerical method, including the physical model, computational method, and mesh generation and verification, is described in Section of Methodology and tools. The unsteady effect and wake propagation for contra-rotating fan are discussed in Section of Analysis of numerical results and discussion. Finally, the conclusions of this work are summarized.

## Methodology and tools

In this section, the contra-rotating fan as well as different configurations surveyed in the paper are discussed. The experimental instrumentation, the numerical method, and the strategy concerning meshing are also discussed.

### Description of the contra-rotating fan configuration

**Fig 1:**
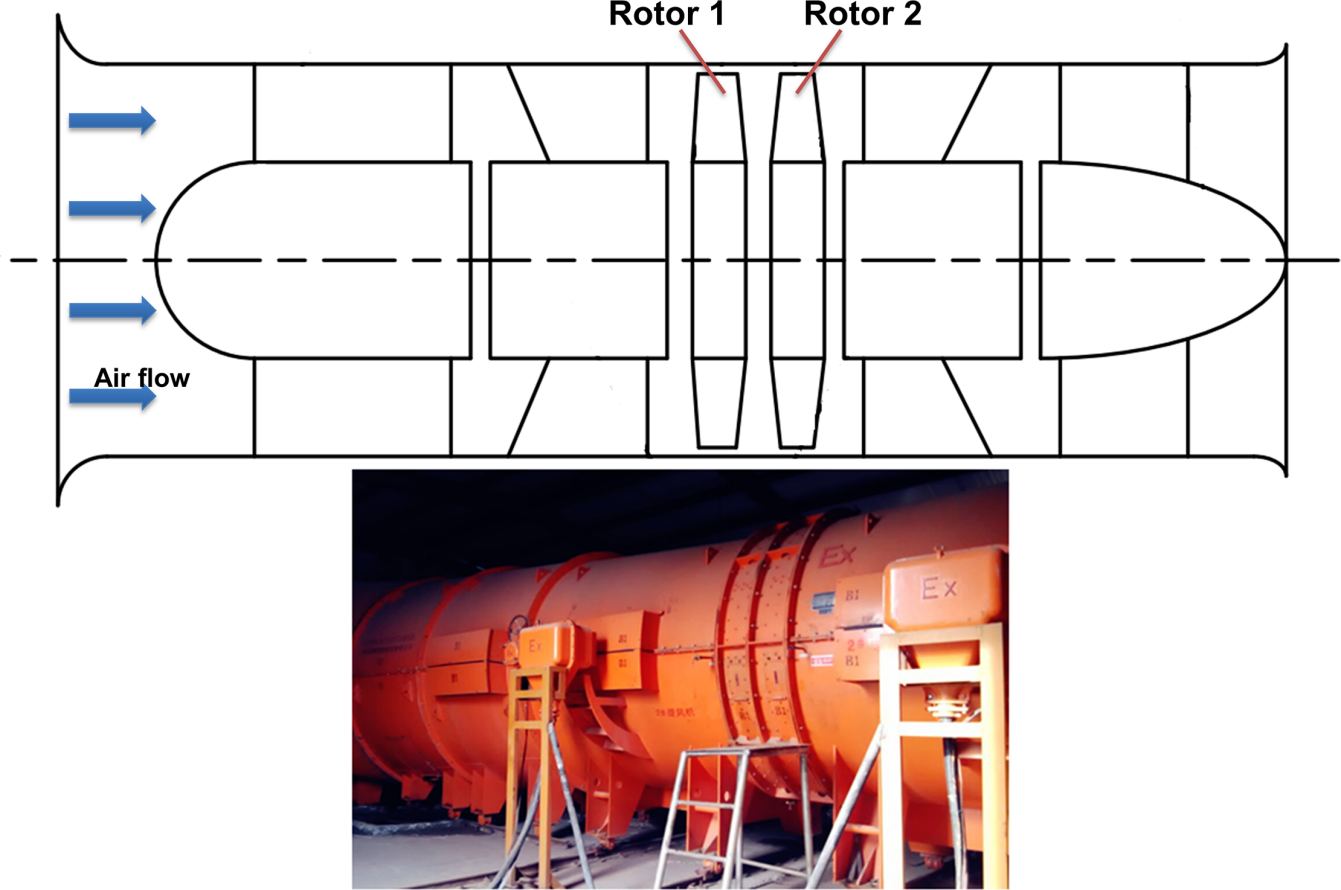
View of contra-rotating fan.

The test case taken into account by the research was a contra-rotating fan involved in the aerodynamic studies. This contra-rotating axial fan, which is also known as FBCDZ-No.20 fan, is shown in Fig 1. It consisted of a clockwise-rotating front rotor (Rotor 1) and an anticlockwise-rotating rear rotor (Rotor 2), with the hub ratio of 0.618. Rotor 1 and Rotor 2 had 19 and 17 blades, and the design speed was -980 rpm and 980 rpm, respectively. This fan was designed at standard atmospheric condition to produce 64m^3^/s airflow. Its detailed aerodynamic design parameters see the literature [8].

For the present study, five contra-rotating fan configurations were investigated, considering different sets of axial spacing dimensions. In general, axial spacing refers to the axial distance between annular edges of two adjacent blades at the average radius position. The original configuration was the experimental configuration corresponding to a contra-rotating fan with nominal axial spacing dimensions (0.3 chord). In addition, designed four kinds of configurations, respectively were 0.5 chord, 0.7 chord, 0.9 chord, 1.1 chord.

### Experimental instrumentation

**Fig 2:**
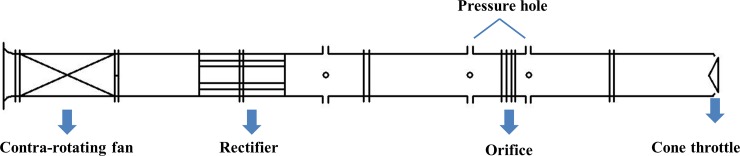
The set-up drawing of out-gas experiment.

For the contra-rotating fan used in the pipeline, according to Chinses GB1236T-2000 regulations, the experiment used the out-gas experiment, that was the experiment of free import and pipeline outlet. The flow and pressure was measured at the outlet. The experimental device used is shown in Fig 2. In flow rate measurement, the in-pipe ISO Venturi nozzle and a matched system for automatic collection and processing of experimental data were used. Experimental devices also included an automatic testing system of aerodynamic performance of a ventilator, involving hardware (sensor, collector and computer) and software. It can be used to measure a series of parameters such as flow rate, total pressure, static pressure and motor power. The orifice differential pressure was measured to calculate the fan flow and the fan duct outlet pressure was used to calculate the fan pressure. The experiment was used to change the inlet flow to measure the other corresponding performance parameters.

### Governing equations and flow solver

Considered from the numerical method in this paper, the governing equations were the unsteady compressible Navier-Stokes equations that describe the conservation of mass, momentum, and energy. It can be expressed in three-dimensional coordinates as

$$\left\{ \begin{array}{l} \frac{\partial \rho }{\partial t}+\frac{\partial (\rho u_{i})}{\partial x_{i}}=0\\ \frac{\partial (\rho u_{i})}{\partial t}+\frac{\partial (\rho u_{i}u_{j})}{\partial x_{j}}=-\frac{\partial p}{\partial x_{i}}+\frac{\partial \tau_{ij}}{\partial x_{j}}\\ \frac{\partial (\rho e)}{\partial t}+\frac{\partial (\rho u_{j}H)}{\partial x_{j}}=\frac{\partial q_{j}}{\partial x_{j}}+\frac{\partial (u_{i}\tau_{ij})}{\partial x_{j}} \end{array}\right.$$ (1)

Where ***u***_*i*_, ***u***_*j*_ are the velocity vectors, *ρ* is the fluid density, *p* is the fluid pressure, *H* is the total enthalpy, *e* is the internal energy, ***τ***_*ij*_ is the viscous stress tensor in Cartesian coordinates, including viscous stress and Reynolds stress, *q*_*j*_ is the heat flux vector.

The classical eddy viscosity assumption was made to compute turbulent fluxes necessary to close the system of URANS equations. A commercial solver package of CFX was utilized for the current work. The flow solver was a coupled pressure-based solver, employed a fully implicit solution strategy. For this study, the time-marching was performed by using an efficient implicit time integration scheme, based on the backward Euler scheme and scalar lower-upper symmetric successive over-relaxation method. This time-marching method was coupled with a second order dual time stepping method to obtain a time consistent solution. The turbulence model with regard to the model of Shear Stress Transport (SST) [21] was applied. It is referred to as a model of SST model which was associated to benefits of *k-ω* turbulence model for simulation of features of the *k-ε* turbulence model and the near-wall boundary layer, wherein small dependence concerning conditions of the far-field boundary was involved. Hence, its accuracy and reliability of prediction are higher [22–24]. The eddy viscosity and *k* equation and *ω* equation of the SST model can be written into the following form:

$$\nu_{t}=\frac{a_{1}k}{\max(a_{1}\omega ;\Omega F_{2})}$$ (2)

$$\frac{D\rho k}{Dt}=\frac{\partial }{\partial x_{j}}\left[(\mu +\sigma_{k}\mu_{t})\frac{\partial k}{\partial x_{j}}\right]+\tau_{ij}\frac{\partial u_{i}}{\partial x_{j}}-\beta^{*}\rho \omega k$$ (3)

$$\frac{D\rho \omega }{Dt}=\frac{\partial }{\partial x_{j}}\left[(\mu +\sigma_{\omega }\mu_{t})\frac{\partial \omega }{\partial x_{j}}\right]+\frac{\gamma }{\nu_{t}}\tau_{ij}\frac{\partial u_{i}}{\partial x_{j}}-\beta \rho \omega^{2}+2(1-F_{1})\rho \sigma_{\omega 2}\frac{1}{\omega }\frac{\partial k}{\partial x_{j}}\frac{\partial \omega }{\partial x_{j}}$$ (4)

Where: *Ω* denotes the vorticity, $F_{2}=\tanh(\arg_{2}^{2})$, $\arg_{2}=\max\left(2\frac{\sqrt{k}}{0.09\omega y};\frac{500\nu }{y^{2}\omega }\right)$, *y* denotes the wall distance. Refer to reference [21] for each closure constant coefficient in the formula.

### Numerical boundary conditions

For practical reasons of computational resources, the whole experimental domain cannot be simulated. As a consequence, we had chosen to use single-passage simulations. With the domain scaling method and under the premise of ensuring the same consistency [25], the number of Rotor 2 was increased from 17 to 19. Rai[26] compared computation results and experimental measurement results of the reduction domain scaling method and pointed out that results of both methods are basically consistent. Arnone[27] verified reliability of applying the reduction domain scaling method in the analysis of rotor-stator interaction. Mayoraca[28] made investigation concerning sensitivity possessed by a technology of domain scaling, involving the prediction of blade forcing and execution of mode excitability. It is shown in research results: estimation effects of the first harmonic excitation concerning the upstream and downstream excitations are good under application of scaling with proper amounts. In addition, estimation of second harmonic quantities is more sensitive to scaling under the case of investigated test.

The material used Air ideal gas. Total pressure, total temperature, and flow angles were given uniformly at the inlet boundary. Averaged static pressure was adopted on the outlet boundary, while radial equilibrium static pressure boundary conditions were specified at the outlet for low mass flow rate working conditions. The adiabatic and non-slip conditions were used for all the surfaces of walls. The steady method used the Mixing-Plane model [29,30], and the unsteady method used the Transient Rotor-Stator model [31,32].

### Mesh strategy

**Fig 3:**
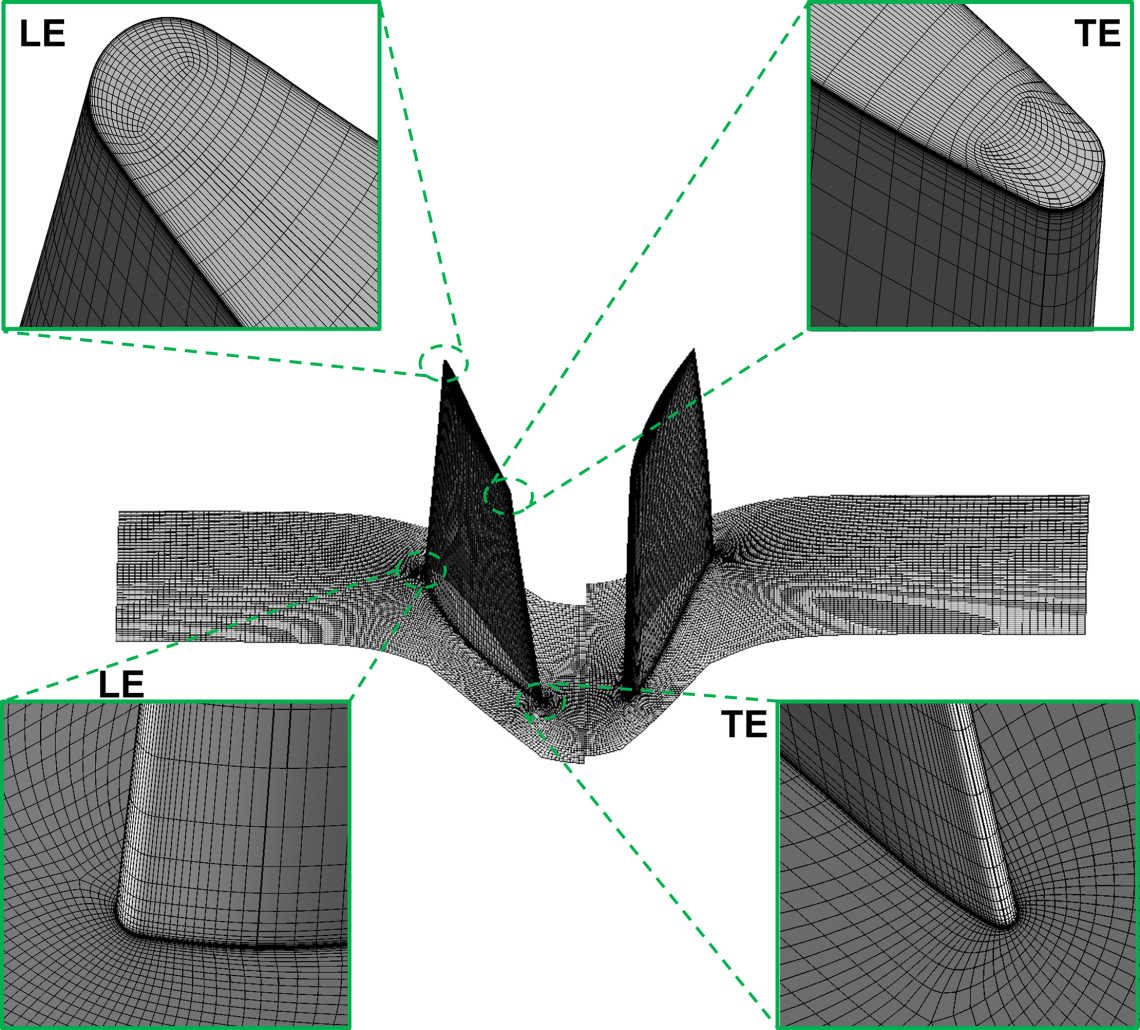
Computational mesh of contra-rotating fan.

As for CFD analysis, the computational mesh is generated by AUTOGRID5 software. The meshes of computation were divided into of the Rotor 1 domain and the Rotor 2 domain. O4H topology was selected for modeling of the major flow passage. Plus, the butterfly topology was used for modeling of the tip clearance of rotors. Quantities of the grid nodes of Rotor 1 and Rotor 2 in stream-wise, span-wise and pitch-wise directions were 123×85×91. The minimum grid spacing on the solid wall was 2×10^-6^m that giving y^+^<2 at the walls. The computational mesh is shown in Fig 3.

**Fig 4:**
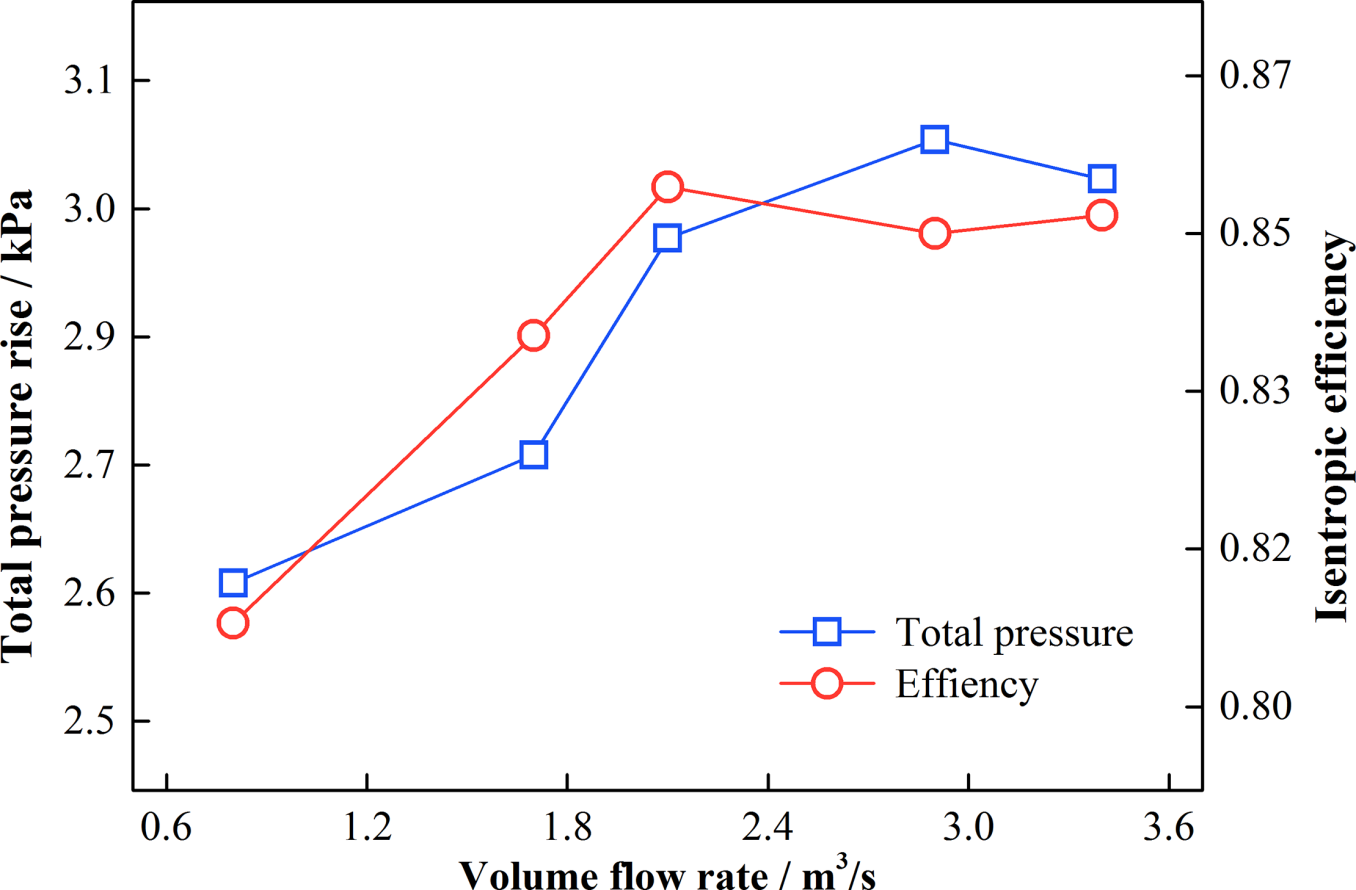
Independent verification of mesh number.

To confirm that the requirements for computational accuracy were satisfied, the gird independence was verified using the contra-rotating fan with different grid numbers. 0.8 million grids were taken as the starting point, and 6 sets of grids were carried out from 0.8 million, 1.7 million, 2.1 million, 2.9 million and 3.4 million respectively. The distribution densities of the 6 different grid nodes were similar, which were mainly used for encryption. The parameters of total pressure and isentropic efficiency were utilized here. As shown in Fig 4, when the mesh number exceeds 2.1 million, the variations of total pressure rise and efficiency are negligible. Hence, a grid number of 2.9 million was applied for the computation under consideration of computational accuracy.

## Analysis of numerical results and discussion

### Validation of numerical method

The present work proposed to simulate the flow in many configurations of a contra-rotating fan. Two effects are investigated in this section, steady RANS and unsteady RANS are compared. Simulation was performed using 32 computing cores of a HP workstation. Unsteady flow simulations star from a steady-state flow solution.

Flow rate and total pressure which were measured are expressed by the non-dimensional parameters. The flow rate coefficient *φ* and the total pressure coefficient *ψ* are computed according to the following formula, respectively:

$$\varphi =\frac{4Q}{\pi D_{t}^{2}U_{t}}$$ (5)

$$\psi =\frac{2P_{t}}{\rho U_{t}^{2}}$$ (6)

Where: *Q* is the volume flow, *P*_*t*_ is the total pressure rise, *D*_*t*_ is the diameter of rotor, *U*_*t*_ is the blade tip linear velocity, *ρ* is the air density.

**Fig 5:**
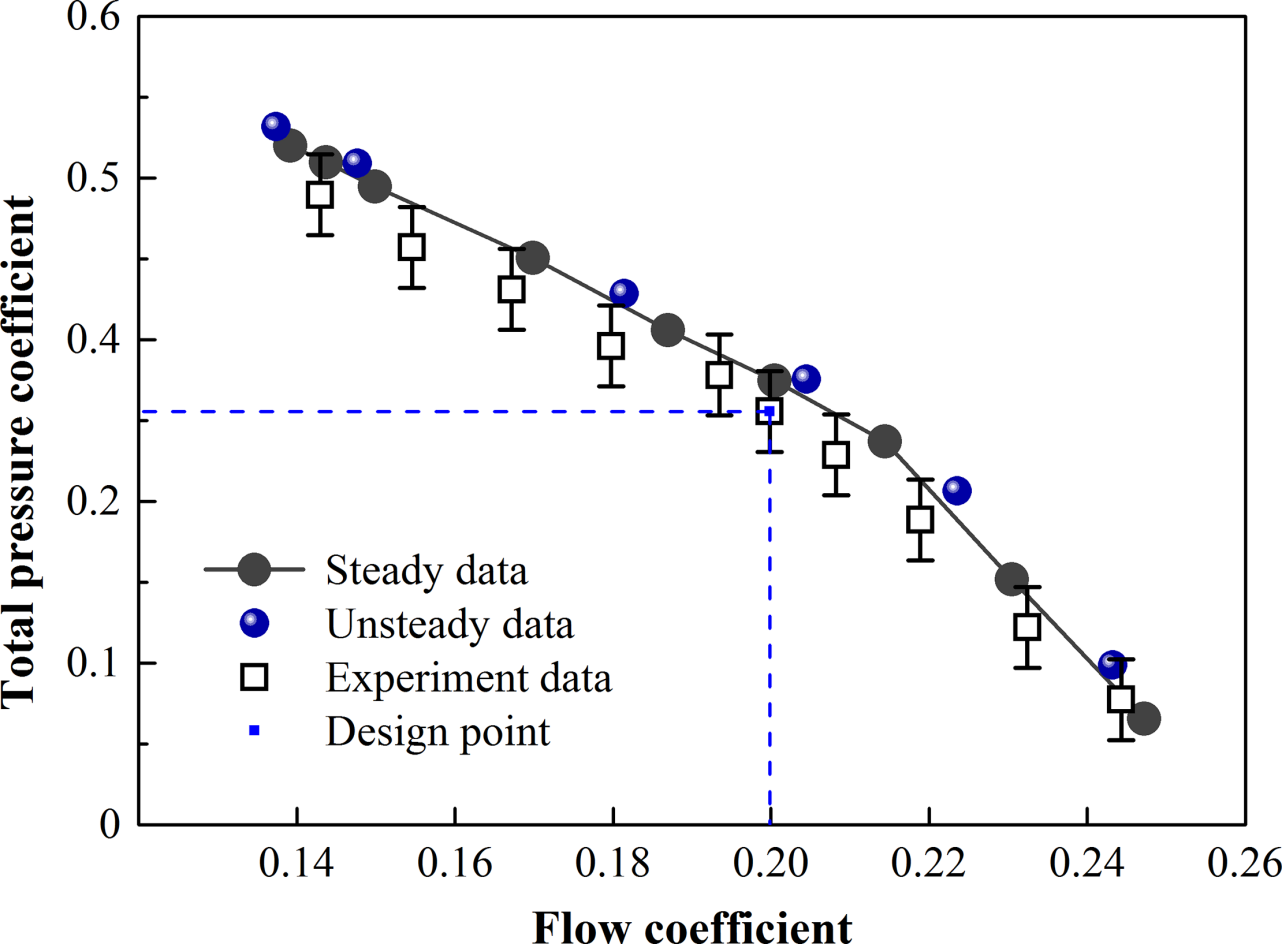
Curve of numerical and experimental total pressure rise performance.

In order to verify the accuracy of numerical results, Fig 5 shows the numerical and experimental total pressure rise performance curve of the contra-rotating fan at the design speed. The stall point was considered as the last operating point before non-convergence. It is feasible to observe the agreement with regard to overall curves of performance between experimental and numerical results in most parts.

### Unsteady effect of two-stages rotors

In order to make researches on the impacts of unsteady effect on two-stage rotors of the contra-rotating fan, loss coefficient $\bar{\varepsilon }$ and relative loss coefficient $\Delta \bar{\varepsilon }$ were introduced. They are defined as follows:

$$\bar{\varepsilon }=\frac{T_{K}(S_{K}-S_{i})}{100C_{p}(T_{K}-T_{i})}$$ (7)

$$\Delta \bar{\varepsilon }={\bar{\varepsilon }}_{steady}-{\bar{\varepsilon }}_{unsteady}$$ (8)

Where: *T*_*K*_ and *T*_*i*_ indicate total outlet temperature and total inlet temperature; *S*_*K*_ and *S*_*i*_ indicate outlet entropy and inlet entropy; $\Delta \bar{\varepsilon }$ indicates the value of steady computation results subtracted by unsteady computation results. The larger value of $\bar{\varepsilon }$ indicates larger flow field losses. When $\Delta \bar{\varepsilon }>0$, the steady loss coefficient is larger than the unsteady loss coefficient, indicating that the unsteady effect could positively control flow losses. When $\Delta \bar{\varepsilon }<0$, the steady loss coefficient is smaller than the unsteady loss coefficient, indicating that unsteady effect deteriorates flow and leads to increase of flow loss.

**Fig 6:**
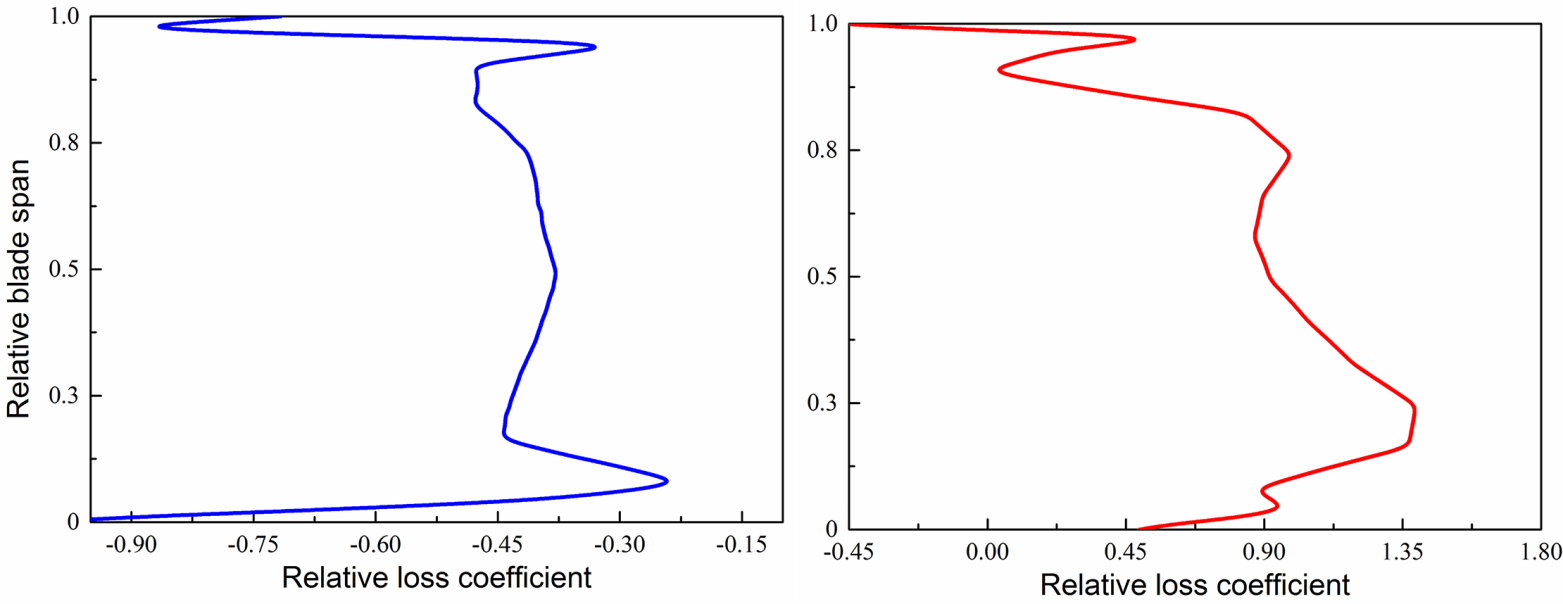
Distribution of relative loss coefficient along blade span of two rotors. (a) rotor 1; (b) rotor 2.

Fig 6 shows a distribution diagram of the relative loss coefficients of two-stage rotors along the blade height direction. The horizontal coordinate indicates the relative loss coefficient. The longitudinal coordinate indicates the relative blade height. Unsteady parameters are time-average values of two cycles. It can be found that relative loss coefficients of rotor 1 were smaller than zero, indicating that unsteady effect enhanced the flow losses. The rotor-rotor interaction borne by rotor 1 was mainly the potential flow interference. The potential flow interference is the unsteady interference which is generated between the blade rows because the pressure field is distributed circumferentially in an uneven manner via the blade channel, so the uneven pressure field is swept by the adjacent blade rows cyclically. Blocking effect caused by the potential flow interference led to increase of flow losses. In addition, relative loss coefficients existed in blade top areas and blade root areas. Strong unsteady flows such as tip leakage vortex and root passage vortex intensified local flow losses.

Relative loss coefficients in most areas of rotor 2 were larger than zero, indicating that unsteady effect positively controlled the overall flow losses of rear stage. Relative loss coefficients of rotor 2 were about twice the absolute values of rotor 1, indicating that unsteady effect had strong influences on rear stage as the flow fields of rear stage would be influenced jointly by upstream wakes and potential flows, while the unsteady effect was stronger. Disturbance propagated to the upstream direction generally has the pressure wave disturbance only. As for entropy disturbance and eddy disturbance, the propagation speed is equal to the local speed of fluid flowing. When it is propagated to the downstream direction, complicated interactions with the downstream secondary flows and boundary layer in the downstream direction will appear during the propagation. In this way, larger unsteady changes will take place in the flow filed.

### Wake propagation analysis

The unsteady effect influences rear stage flow fields greatly and can control flow losses. In the contra-rotating fan, upstream wakes periodically sweep rear flow passages and have important impacts on rear flow fields. Hence, this paper will then analyze unsteady effect of wakes.

Smith [33] proposed the following simple model which is based on wake lengths for the recovery of total pressure mixing loss.

$$R=1-{\left(\frac{L_{in}}{L_{exit}}\right)}^{2}$$ (9)

Where: *L*_*in*_ and *L*_*exit*_ indicate lengths of inlet and outlet wakes. When the wakes are extended more in the blade channel, the length ratio of inlet and outlet wakes would be smaller and value of *R* would be larger. The stronger the recovery of total pressure mixing loss is, the smaller the losses would be. A lot of scholars [3, 34] verified and developed wake theories, indicating that wakes could improve aerodynamic performance of rotors under certain conditions. However, wake recovery effects of contra-rotating fans were rarely researched in a profound manner.

**Fig 7:**
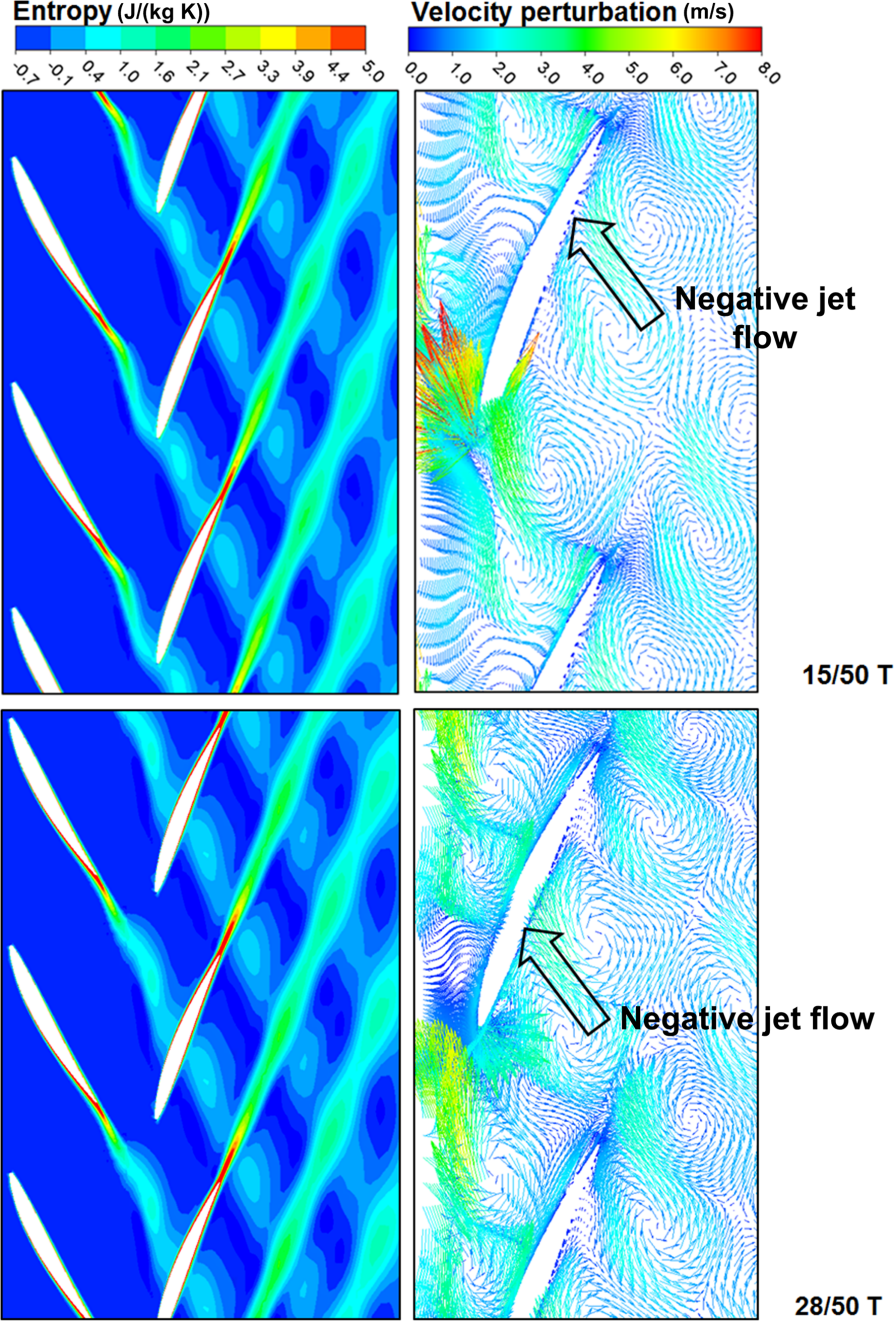
Contours of entropy and velocity perturbation vectors.

In fan/compressor under multi-stage environments, velocity losses of wakes and strong turbulence intensity in wakes would have strong interference on downstream blades, leading to significant unsteady effect of downstream flow fields. Fig 7 shows an entropy contour and a velocity perturbation vector diagram of 50% blade height cross sections at two typical moments. The velocity perturbation vector is the sum of perturbation component velocities of different directions. Specific definition is as follows:

$$\vec{v_{p}}=\vec{v_{x}}+\vec{v_{y}}+\vec{v_{z}}$$ (10)

Where: $\vec{v_{x}}$ denotes axial perturbation velocity; $\vec{v_{y}}$ denotes tangential perturbation velocity; $\vec{v_{z}}$ denotes radial perturbation velocity. The perturbation component velocity is the difference between transient velocity in each direction and the time average velocity, reflecting pulsation of airflow velocities.

Entropy values of wake areas were much larger than those of main flow areas, constituting major losses. During downward propagation of upstream wakes, the wakes were cut by the rear rotor and got stretched and deformed in the rear blade passage. Through observation of wake transport, we can find that at the moment of 15/50 T, upstream wakes exactly reached the leading edge of rear blade. With contra rotation of two-stage rotors, the upstream wakes were already cut by the rear blade at the moment of 28/50 T. During the whole course when upstream wakes flowed to the downstream areas, adverse pressure gradients led to expansion and stretching of wakes in the rear blade passage. Influence area of wakes were expanded. They got blended with main flows. Influenced by upstream wakes, the flow high-loss areas were gradually reduced along the flow direction. It is shown in the diagram that outlet wakes were obviously lengthened in the blade passage. In combination with above wake model, we could find that wake recovery effect in the contra-rotating fan was strong. Hence, flow losses of the rear rotor were reduced.

According to vector diagram of perturbation velocities, we can find that, as influenced by upstream wakes, perturbation vortex pairs with the opposite rotation directions appeared in the blade passage of rear rotor. Influenced by pressure gradients, the motion direction of perturbation vortex pairs was opposite to the blade rotation direction and gradually got deviated to the blade pressure surface. During motion, viscous shear effect took place between perturbation vortex pairs and main flows, leading to flow losses. The disturbance speed depends on loss degree of wakes. The more serious losses the wake has, the larger difference will be between the relative speed in the wake and that of the main flow. Hence, the generated disturbance speed will be larger. In addition, the negative jet flow phenomenon could be observed in the rear flow passage, as presented by black frame arrows in Fig 6. Negative jet flows appeared as obvious velocity losses of fluids inside the wakes existed relative to main flows, so that the pulsation velocity directions of fluids inside wakes directed to the blade pressure surface. When negative jet flows moved to the pressure surface of rear stage blade, negative jet flows increased energy on boundary layers of the blade, and the boundary layers were more capable of overcoming adverse pressure gradients. Hence, flow separation was delayed. When fluids inside wakes flowed out, high-energy main flows kept on flowing into wakes. Kinetic energy of wake areas was enhanced. The wake recovery factor was increased. Hence, the negative jet flow phenomenon also enhanced wake recovery effect, leading to decrease of flow losses of the rear rotor.

### Influence of axial spacing on unsteady effect

**Fig 8:**
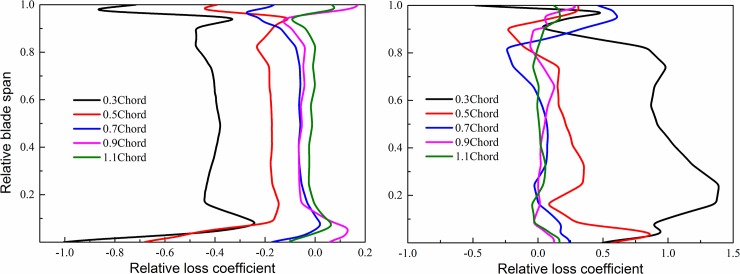
Distribution of relative loss coefficient along blade span of two rotors with respect to different axial spacing. (a) rotor 1; (b) rotor 2.

Distribution of relative loss coefficients of rotor 1 and rotor 2 along the blade height direction under different axial spacing is shown in Fig 8. As a whole, the axial spacing had significant influences on unsteady effect of two stages of rotors. With the increase of axial spacing, the absolute values of relative loss coefficients gradually decreased and approached 0, indicating that unsteady interference effect between two stages of rotors were obviously weakened with the increase of axial spacing.

As for the rotor 1, relative loss coefficients were negative, indicating that unsteady effect always had negative influences on front stage flow losses with changing of axial spacing. We can see that the relative loss coefficient decreased rapidly with axial spacing. When axial spacing was larger than 0.7 chord, it has little effect on the unsteady effect. On the contrary, the unsteady effect was very sensitive to axial spacing in the range of 0.3 chord-0.7 chord. Under the axial spacing of 0.3 chord, the absolute value of relative loss coefficient was maximum. With the increase of axial spacing, the absolute value of relative loss coefficient under 0.5 chord was obviously reduced. It was reduced by a half relative to the situation under 0.3 chord. Under 0.7 chord, the absolute value of relative loss coefficient further decreased. At this moment, unsteady effect only had very limited influences on flow losses. Under 1.1 chord, the absolute value of relative loss coefficient basically approached 0, indicating that under the large axial spacing, the downstream potential perturbation had very tiny influences on the rotor 1, while unsteady effect only had slight effect on front stage flow losses.

As for the rotor 2, with the changing of axial spacing, unsteady effect basically had positive effects on control of flow losses. In comparison with the rotor 1, the relative loss coefficient of rotor 2 was more sensitive to changing of the axial spacing. When axial spacing was larger than 0.5 Chord, it has little effect on the unsteady effect. Under the axial spacing of 0.3 chord, the relative loss coefficient was maximum. When the axial spacing increased to 0.5 chord, the relative loss coefficient decreased greatly. When the axial spacing ranged from 0.7 chord to 1.1 chord, the relative loss coefficient of rotor 2 was basically zero. The results indicates that under large axial spacing, unsteady effect only had very slight influences on flow losses of the rotor 2, while interaction effect between two-stage blade rows could not provide gains for unsteady efficiency of the rotor 2 anymore.

**Fig 9:**
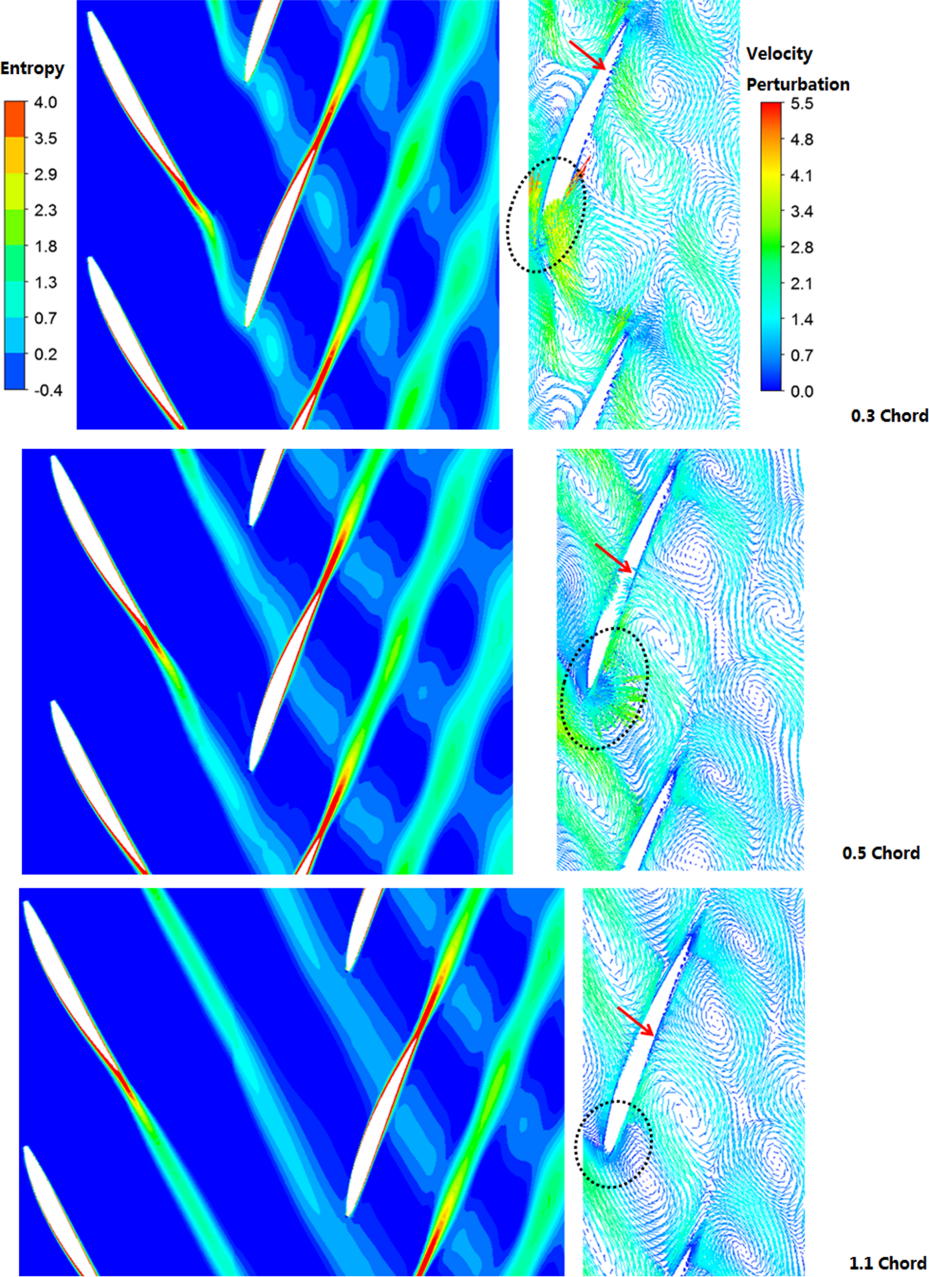
Contours of entropy and velocity perturbation vectors with respect to different axial spacing.

In order to clearly observe propagation courses of upstream wakes under different values of axial spacing, Fig 9 shows entropy contours and velocity perturbation vector diagrams at the same transient moment under different axial spacing. Cross section of 50% relative blade height was selected as the blade to blade section. Flow forms of wakes show that vibration amplitudes of wakes decreased with the increase of axial spacing. Under the axial spacing of 0.3 chord, the wake vibration characteristics were very strong. With the increase of axial spacing, wake vibration characteristics basically disappeared, reflecting weakening of potential perturbation effect of the rear rotor.

Length of the perturbation velocity vector denotes size of the pulsation velocity. It is shown in the diagram that, under the axial spacing of 0.3 chord, the leading edge of rotor 2 was impacted by upstream wakes and showed violent speed perturbation. As shown by black dotted lines in the diagram, the perturbation velocity on leading edge of the rotor 2 was obviously weakened under 0.5 chord. Under the large axial spacing (1.1 chord), perturbation velocity intensities of the whole blade of rotor 2 were consistent. At this moment, effects of wakes on the leading edge of blade were not obvious. This result indicates that increase of the axial spacing could reduce airflow attack angles of the blade of rotor 2, so that flow field situations of the rotor 2 could be improved. In addition, we could find that during propagation of upstream wakes to downstream areas, negative jet flows existed in the rear blade row passage, as presented by red arrows in the diagram. It is concluded in Section 4.2 that the negative jet flow phenomenon could enhance boundary layer energy to a certain extent and postpone flow separation. Under the axial spacing of 0.3 chord, the negative jet flows approached the trailing edge of blade and roughly reached the position of 85% chord length blade. When the axial spacing increased, negative jet flows moved forward to the position of 50% blade chord length of the blade, while fluid energy of negative jet flows were weakened obviously, indicating that negative jet flows were significantly influenced by the axial spacing. Under the small axial spacing, wakes wall give rise to a larger disturbance speed and stronger negative jet flow effect. When the negative jet flow effect gradually increases, the wakes will continuously migrate and gather to the pressure surface. Therefore, length of the wakes will be shortened, while recovery effect of the wakes will be stronger. Hence, under the small axial spacing, the unsteady effect in rotor 2 played a significantly positive role in control of flow losses.

### Unsteady pressure characteristics on blade surface

The root mean square (RMS) static pressure can reflect the area where unsteady flows occur, and it can also reflect the fluctuation intensity of the pressure on the blade surfaces. The RMS static pressure is defined as below:

$$P_{RMS}=\frac{1000}{\bar{p}}\sqrt{\frac{1}{T}\int_{0}^{T}\left[p(z,r,\theta ,t\right){]}^{2}dt}$$ (11)

Where: *p* denotes a unsteady value of pressure pulsation in flow fields; $\bar{p}$ denotes a time-average pressure of the flow field; *T* denotes a unsteady time cycle.

**Fig 10:**
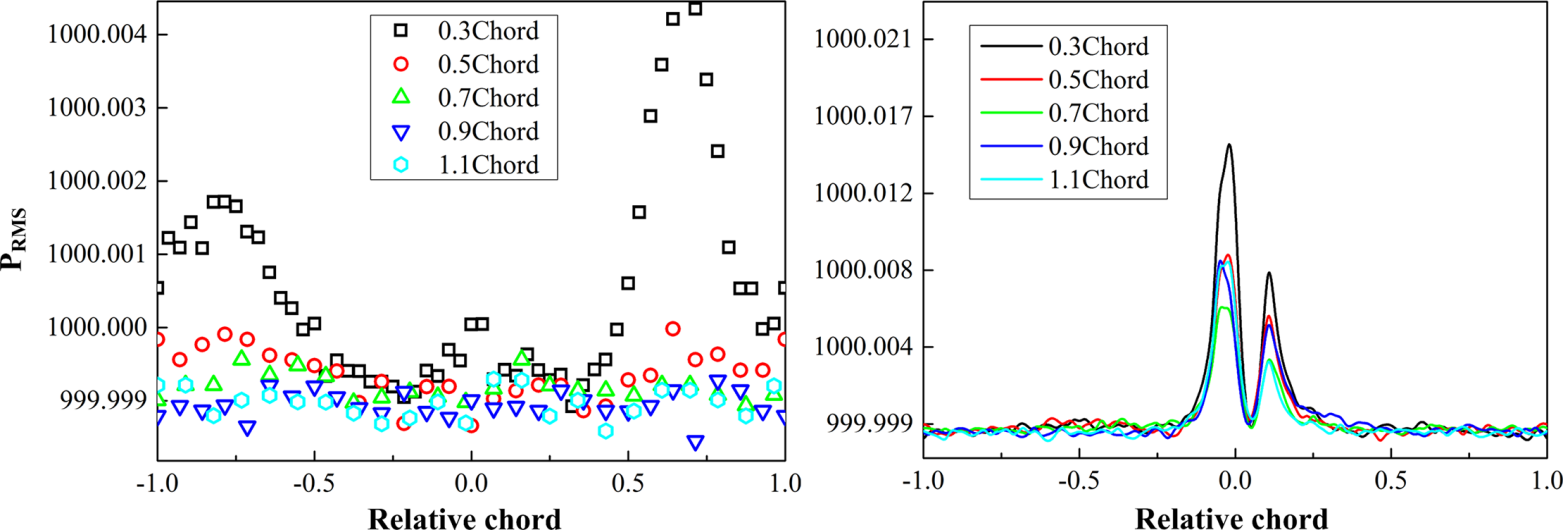
RMS pressure of two rotors with different axial spacing. (a) rotor 1; (b) rotor 2.

This paper used the RMS pressure distribution to evaluate the unsteady characteristics of the contra-rotating rotors. Fig 10 shows RMS pressures of two-stage rotors with different axial spacing on three typical blade height cross sections, wherein the longitudinal coordinates denote RMS pressures, while horizontal coordinates denote relative chord lengths of blades. Negative relative chord length indicates blade suction surface, positive relative chord length indicates blade pressure surface.

As a whole, with the increase of axial spacing, the unsteady fluctuation strength of static pressure on the blade surface was reduced significantly, while the position of maximum fluctuation strength was deflected. As for the rotor 1, obvious RMS pressure fluctuation peaks appeared in the blade root area under the axial spacing of 0.3 chord. They were located at positions of 25% and 75% of relative chord lengths on the pressure surface as well as positions close to the trailing edge on the suction surface. When the axial spacing increased to 0.5 chord, the RMS pressure fluctuation degree was decreased significantly, while the RMS pressure fluctuation amplitudes became very weak after 0.9 chord. In the blade middle area and blade top area of the rotor 1, the RMS pressure fluctuation was most obvious under the axial spacing of 0.3 chord, wherein fluctuation peaks were located at the leading edge of blade, 75% relative chord length of the pressure surface and the position on the suction surface close to the trailing edge. Under the axial spacing of 0.5 chord, RMS pressure fluctuation peaks disappeared at the rotor 1; fluctuation peaks were located at the 70% relative chord length of the pressure surface and the trailing edge of the suction surface, respectively; the amplitude decreased obviously. After the axial spacing of 0.9 chord, the RMS pressure fluctuation peaks disappeared.

As for the rear rotor, two peaks appeared on RMS pressure fluctuation curves at each blade height cross sections and were located at the suction surface of the blade leading edge and the pressure surface side, respectively, wherein the maximum fluctuation amplitude appeared at 0.3 chord. With the increase of axial spacing, the RMS pressure fluctuation peak decreased. But after the axial spacing of 0.5 chord, the reduction degree of the RMS pressure fluctuation amplitude was not obvious.

### Rotor-rotor interactions on aerodynamic force on blade surface

**Fig 11:**
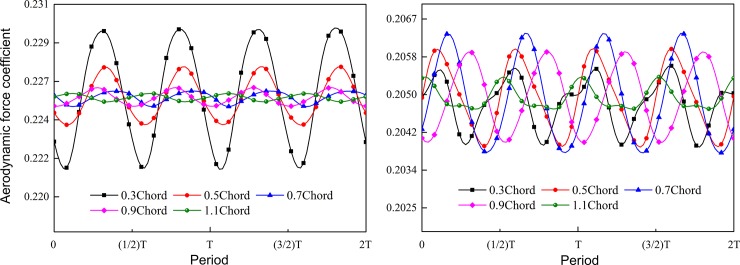
Blade aerodynamic force fluctuation with different axial spacing. (a) rotor 1; (b) rotor 2.

Fig 11 shows the diagrams of aerodynamic force fluctuation which acts on two-stage rotor blades during two computation cycles, wherein the horizontal coordinate denotes a relative cycle, and the longitudinal coordinate denotes the aerodynamic force coefficient. The aerodynamic force of the blade is integrated by static pressure on the blade surface:

$$\begin{array}{l} F=\sqrt{F_{x}{}^{2}+F_{y}{}^{2}+F_{z}{}^{2}}\\ F_{x}=\oint_{\Omega }P\vec{n}\cdot \vec{u}dA\\ F_{y}=\oint_{\Omega }P\vec{n}\cdot \vec{v}dA\\ F_{z}=\oint_{\Omega }P\vec{n}\cdot \vec{w}dA \end{array}$$ (12)

Where, *p* denotes static pressure on the blade surface, $\vec{n}$ denotes the outer normal unit vector on the blade surface, $\vec{w}$, $\vec{v}$ and $\vec{u}$ denote axial, tangential and radial unit vectors, and *Ω* denotes the blade surface.

The blade aerodynamic force is expressed by the non-dimensional parameters. The aerodynamic force coefficient is computed according to the following formula:

$$C_{F}=\frac{F}{\rho \pi hL_{h}{\left(r_{m}\omega \right)}^{2}}$$ (13)

Where: *h* is the blade height, *L*_*h*_ is the hub axial length, *ω* is the rotational speed and *r*_*m*_ is the mean radius of the blade.

Fig 11(A) displays an unsteady aerodynamic force fluctuation diagram of Rotor 1. It is shown in the diagram that unsteady aerodynamic force of Rotor 1 was affected obviously by axial spacing; with increase of axial spacing, the fluctuation amplitude of aerodynamic force decreased, but time-average values of aerodynamic force were not affected by the axial spacing obviously. Under the axial spacing of 0.3 chord, aerodynamic force of Rotor 1 blade had the largest fluctuation amplitude of about 19N, under the axial spacing of 1.1 chord, the fluctuation amplitude of aerodynamic force was only 1N, indicating that under the large axial spacing, effects of downstream potential disturbance on aerodynamic force of Rotor 1 could be ignored. Under different axial spacing values, time-average values of aerodynamic force coefficient of the Rotor 1 blade were basically equal to 0.2256.

Fig 11(B) displays an unsteady aerodynamic force fluctuation diagram of Rotor 2. It is shown in the diagram that axial spacing only had very small effects on time-average value of unsteady aerodynamic force of blades, and the time-average value of aerodynamic force coefficient of blades under different axial spacing values was about 0.2049. Fluctuation amplitudes of aerodynamic force were affected by axial spacing, but affecting rules were different from those of Rotor 1. Fluctuation amplitude of unsteady aerodynamic force of Rotor 2 blade increased first and then decreased with the increase of axial spacing. Under the axial spacing of 0.3 chord, the aerodynamic force fluctuation amplitude was 4.2N, under 1.1 chord, the fluctuation amplitude reached the value of 1.6N. The aerodynamic force fluctuation of Rotor 2 was mainly caused by upstream potential flow disturbance and upstream wakes, and under large axial spacing, the influence of potential flow disturbance and upstream wakes on the aerodynamic force fluctuation of Rotor 2 blade was significantly reduced.

In conclusion, relative motion of the two-stage rotors make the wake and potential flow interference exert strong influences on the unsteady characteristic of the flow field, and thus the aerodynamic force of blade changed subsequently. With the increase of axial spacing, the rotor-rotor interaction effect of two-stage rotors was weakened, while the fluctuation amplitude of the blade aerodynamic force decreased. When the axial spacing reached 0.5 chord and kept increasing, the changing degree of unsteady effects in the rotors will be negatively correlate. In addition, Reference [9] points out that the contra-rotating fan has the highest efficiency under the 0.5 chord. Therefore, based on comprehensive analysis of performance in terms of fan dynamic forces and efficiency, the optimum axial spacing for contra-rotating fan is 0.5 chord. In the future aerodynamic design, the aerodynamic performance of fan can be further optimized through analysis of the flow field structure in combination with the numerical optimization technology. The optimization design method is mainly based on the idea that the optimization direction of aerodynamic design can be obtained by the optimization control theory in a vector space constituted of designed parameters [35,36]. The optimization method will play a more and more important role in future aerodynamic optimization design of fans.

## Conclusions

The reported idea of unsteady effect and wake propagation for contra-rotating fan is studied. The work also shows results of numerical simulations for five configurations of the same contra-rotating fan. The original configuration corresponds to the experimental version while other configurations consider the increasing axial spacing. The following conclusions can be drawn from the initial studies:

1. The rotor-rotor interaction in the contra-rotating fan played an important role in aerodynamic efficiency of rotors. Unsteady effect increased flow losses of the rotor 1, but effectively inhibited flow losses of the rotor 2. With the increase of axial spacing of two rotors, the effect of rotor-rotor interaction between two rotors decreased, while unsteady effect of local flow fields was weakened. When the axial spacing increased to 1.1 chord, unsteady effect only had very tiny influences on aerodynamic efficiency of rotors.
2. In comparison with the rotor 1, unsteady effect had great influences on flow fields of the rotor 2 mainly because of inhibition effect of upstream wakes on separation. The inhibition effect was mainly caused by wake recovery effect of upstream wakes in the rear stage flow passage. Meanwhile, negative jet flows enhanced boundary layer energy of the rear blade. The boundary layers were more capable of overcoming adverse pressure gradients, so that rear flow separation was postponed. With the increase of axial spacing, the acting positions of negative jet flows on the blade were moved towards the leading edge of blade, while fluid energy in negative jet flows was obviously weakened as well.
3. In terms of blade aerodynamics, the influence of the downstream rotor potential flow disturbance on the blades of rotor 1 is greater than the effect of the upstream rotor wake on the blades of rotor 2. Axial spacing had very small effects on average values of unsteady aerodynamic force of two-stage rotors and had large effects on fluctuation amplitudes of aerodynamic force. With the increase of axial spacing, fluctuation amplitudes of aerodynamic force of rotor 1 decreased, and fluctuation amplitudes of aerodynamic force of rotor 2 increased at first and then decreased.
4. The rotor-rotor interaction leads to turbomachinery characterized by very complicated flow patterns, such as swirling flows and breakdowns of large-scale vortical structures. The subject of upstream/downstream nonlinear blade pass interaction requires accurate prediction of acoustic pressure waves. Large-eddy simulation (LES) is a turbulence numerical simulation method between Direct Numerical Simulation (DNS) and Reynolds-averaged Navier-Stokes (RANS). It is used for direct simulation of unsteady motion of large-scale eddies in the turbulence. In the future work, the Large Eddy Simulation will be used to research this problem.

## Supporting information

S1 File Data for Fig 5 (.XLSX); Fig 6 (.XLSX); Fig 8 (.XLSX); Fig 10 (.XLSX); Fig 11 (.XLSX). (ZIP)
